# The Prevalence and Pattern of Using Complementary and Alternative Medicine in Saudi Patients With Diabetes: A Cross-Sectional Study

**DOI:** 10.7759/cureus.30700

**Published:** 2022-10-26

**Authors:** Mohammed Z Aljulifi, Fahad Alfhaid, Awad Alshahrani, Khawlah A Albatil, Raseel A Aljthalin, Farah Alloboon, Raneem Abdulaziz Aljthalin, Arwa S Aljagwani, Dareen A Alenzi

**Affiliations:** 1 Family and Community Medicine, Majmaah University, Majmaah, SAU; 2 Endocrinology, College of Medicine, King Saud Bin Abdulaziz University for Health Sciences, Riyadh, SAU; 3 Medicine, Ministry of National Guard Health Affairs, King Abdullah International Medical Research Center, Riyadh, SAU; 4 Family Medicine, King Saud Medical City, Riyadh, SAU; 5 Neurology, Prince Sultan Military Medical City, Riyadh, SAU; 6 Family Medicine, Prince Sultan Military Medical City, Riyadh, SAU; 7 Emergency Medicine, King Saud Medical City, Riyadh, SAU; 8 Internal Medicine, King Fahad Medical City, Riyadh, SAU

**Keywords:** saudi arabia, cross-sectional study, herbal medicine, diabetes mellitus, complementary and alternative medicine

## Abstract

Background

Diabetes mellitus is a common disease in Saudi Arabia. Patients with chronic diseases, such as diabetes, tend to use complementary and alternative medicine (CAM) either as an addition or alternative to their medical therapy. Many studies have evaluated the CAM herbal products used by patients with diabetes; however, there have been few and inconsistent studies on other types of CAM, and most studies on CAM have focused on their use in type 2 diabetes.

Objective

This study aimed to determine the prevalence and patterns of CAM use among patients with type 1 or type 2 diabetes in Riyadh, Kingdom of Saudi Arabia.

Methods

This cross-sectional study was conducted in an adult Saudi population at King Abdulaziz Medical City, Riyadh, Kingdom of Saudi Arabia. Data were collected from December 2019 to February 2020 using a data collection form and patient interviews.

Results

We included 332 patients, 43% of whom had type 1 diabetes; 26% of the patients had previously used one or more types of CAM. Approximately 53% of CAM users had glycated hemoglobin (HbA1c) level of ≥9%. Among CAM users, 51% mentioned that their blood sugar readings were improved with CAM treatments. Mind-body therapy was the most commonly used CAM (54%), followed by biologically based CAM, including herbs (46%). The most commonly used herbal supplements were black cumin (42%), followed by fenugreek (28%), myrrh (24%), frankincense (22%), cinnamon (15%), garlic (15%), and onion (15%). Older age and employment status were predictors of CAM use in Saudi patients with diabetes. The main sources of knowledge about CAM were from family and friends.

Conclusions

CAM use is common among Saudi Arabian patients with diabetes. Patients with diabetes who are aged >65 years and employment status were the main predictor of CAM use. Assessing CAM use is an important aspect of clinical encounters with Saudi patients, especially patients with type 1 diabetes.

## Introduction

Diabetes mellitus is a chronic disease that requires patients to use medications and other interventions over a long period. The global prevalence of diabetes among adults (aged 20-79 years) will increase by 7.7%, approaching 439 million adults, by 2030. Between 2010 and 2030, a 69% increase in the number of adults with diabetes in developing countries and a 20% increase in developed countries is expected [1]. According to a Saudi health information survey, in collaboration with the University of Washington, the total prevalence of diabetes was 14.8% for males and 11.7% for females in 2013 [2].

Although complementary and alternative medicine (CAM) are different terms, they are mostly used interchangeably [3]. These terms describe an intervention that is used either in addition or as an alternative to standard medical treatment, including, but not limited to, pharmacological and physical remedies and dietary approaches [4]. Chronic diseases are associated with increased use of CAM among patients [5-7]. In 2007, approximately $33.9 billion was spent by adult Americans on CAM therapies [8]. A meta-analysis published in 2012 found that the prevalence of CAM use internationally ranged from 9.8% to 76% [9]. In Saudi Arabia, the use of CAM therapy is estimated to be 46%-68% for all diseases [10,11]. In one survey, 74% of patients had visited CAM providers within the previous 12 months, and the percentage decreased to 47.6% when spiritual healers were excluded. The estimated annual expenditure on CAM was 327,970 Saudi riyals [7]. In 2014, a cross-sectional study assessing the use of herbal medicine among the Saudi population found that 88.4% of participants had used herbal medicines, and most had used them for therapeutic reasons, with a success rate of 61.2% [12].

Therefore, this study aimed to determine the prevalence and pattern of CAM use among patients with diabetes, especially patients with type 1 diabetes, in Riyadh.

This article was previously posted in the Journal of Medical Internet Research (JMIR) Preprints on October 28, 2021.

## Materials and methods

Study sample

This cross-sectional study targeted the adult Saudi population (aged 18 years and above) who were previously diagnosed with any type of diabetes and visited the adult diabetes and endocrine clinic at King Abdulaziz Medical City (KAMC), Riyadh, Kingdom of Saudi Arabia, between December 2019 and February 2020. We used a convenience sampling method in which every adult patient with diabetes who attended the clinic was recruited as part of this study. Adolescents and children with diabetes, patients who declined to participate or did not complete the interview, and pregnant women with gestational diabetes mellitus were excluded from the study.

Data collection

Data were collected by trained medical interns through a data collection form after interviewing the patients. The form contained three parts. The first section collected personal data, the second covered the patient’s diabetes history, and the last assessed CAM use, the type of CAM used, and the effect of CAM use on diabetes. The dependent variable was the use of CAM; independent variables included age, sex, type of diabetes, duration of diabetes, complications of diabetes, and HbA1c. We measured the use of CAM in patients with type 1 diabetes and determined the relationship between glycated hemoglobin (HbA1c) levels and control of the blood glucose levels with the use of CAM.

Each form had a unique identifier. Quality control was applied through the double entry of data.

Statistical analysis

Data were analyzed using SAS version 9.4 (SAS Institute Inc., Cary, NC, USA), a statistical software. Descriptive statistics were used to describe categorical variables. The chi-square test and Fisher’s exact test were used to determine the association between categorical variables. All results were considered statistically significant at P<0.05.

Ethical considerations

Informed consent was obtained from all patients. All human rights were respected by ensuring privacy and no potential harm, and all information was kept confidential. Ethical approval was obtained from the Institutional Review Board of King Abdullah International Medical Research Center with reference number RC19/360/R.

## Results

We interviewed 377 patients, of whom 45 declined to participate, leading to a response rate of 88% (n=332). Approximately 60% of the patients were female. Of them, 25% had attained high-school level education and 45% had a university degree. Patients with type 1 diabetes accounted for 43% of the sample. Table 1 provides more details about patient characteristics. Of the 332 patients, 26% had used some type of CAM. Of the patients with type 1 diabetes, 33% had used a type of CAM. Approximately 53% of CAM users had an HbA1c level of ≥9%; however, 62% of non-CAM users had significantly lower HbA1c levels (≤8%) (P=0.0055).

**Table 1:** Patient characteristics (n=332) *Chi-square test **Fisher’s exact test HbA1c: glycated hemoglobin; CAM: complementary and alternative medicine

Variable	Total (n (%))	CAM user (n=89) (n (%))	Non-CAM user (n=252) (n (%))	P-value
Age (years)				0.0063*
18 to <65	303 (91.27)	67 (83.75)	236 (93.65)	
≥65	29 (8.73)	13 (16.25)	16 (6.35)	
Sex				0.5917*
Female	199 (59.94)	50 (62.50)	149 (59.13)	
Male	133 (40.06)	30 (37.50)	103 (40.87)	
Education level				0.0775*
Illiterate	36 (10.84)	12 (15.00)	24 (9.52)	
Primary/can read and write	42 (12.65)	16 (20.00)	26 (10.32)	
High school	83 (25)	19 (23.75)	64 (25.40)	
University education	149 (44.88)	29 (36.25)	120 (47.62)	
Postgraduate	22 (6.63)	4 (5.00)	18 (7.14)	
Employment status				0.0353*
Employee	97 (29.22)	14 (17.50)	83 (32.94)	
Retired	58 (17.47)	16 (20.00)	42 (16.67)	
Student	69 (20.78)	16 (20.00)	53 (21.03)	
Unemployed	108 (32.53)	34 (42.50)	74 (29.37)	
Marital status				0.1238*
Divorced	12 (3.61)	3 (3.75)	9 (3.57)	
Married	193 (58.13)	51 (63.75)	142 (56.35)	
Single	106 (31.93)	18 (22.50)	88 (34.92)	
Widow	21 (6.33)	8 (10.00)	13 (5.16)	
Diabetes type				0.0284*
Type 1 diabetes	143 (43.07)	26 (32.50)	117 (46.43)	
Type 2 diabetes	189 (56.93)	54 (67.50)	135 (53.57)	
Duration since diagnosis (years)				0.9979*
1-3	25 (7.53)	6 (7.50)	19 (7.54)	
3-10	102 (30.72)	25 (31.25)	77 (30.56)	
10-20	115 (34.64)	27 (33.75)	88 (34.92)	
>20	90 (27.11)	22 (27.50)	68 (26.98)	
Types of treatment				
Oral or injectable hypoglycemic agents	151 (44.28)	43 (48.31)	108 (42.86)	0.3729*
Insulin injections	231 (67.74)	51 (57.30)	180 (71.43)	0.0143*
Diabetes complication				
Nephropathy	36 (10.56)	7 (7.87)	29 (11.51)	0.3364*
Retinopathy	69 (20.23)	20 (22.47)	49 (19.44)	0.3364*
Neuropathy	58 (17.01)	17 (19.10)	41 (16.27)	0.5411*
Cardiovascular disease (stroke, myocardial infarction, heart failure)	43 (12.61)	9 (10.11)	34 (13.49)	0.4090*
Diabetic foot	42 (12.32)	17 (19.10)	25 (9.92)	0.0235*
Never been diagnosed with any complications	206 (60.41)	47 (52.81)	159 (63.10)	0.0880*
Associated comorbidities				
Hypertension	121 (35.48)	41 (46.07)	80 (31.75)	0.0152*
Dyslipidemia	157 (46.04)	42 (47.19)	115 (45.63)	0.8001*
Thyroid disorder	48 (14.08)	19 (21.35)	29 (11.51)	0.0217*
Other	7 (2.05)	2 (2.25)	5 (1.98)	0.8804**
I do not have any of the mentioned diseases	113 (33.14)	20 (22.47)	93 (36.90)	0.0129*
Smoking status				0.2722*
Ex-smoker	30 (9.04)	9 (11.25)	21 (8.33)	
Non-smoker	277 (83.43)	68 (85.00)	209 (82.94)	
Current smoker	25 (7.53)	3 (3.75)	22 (8.73)	
HbA1c				0.0055*
<7 to 8	188 (58.2)	37 (47.44)	151 (61.63)	
9-10	87 (26.93)	32 (41.03)	55 (22.45)	
>10	48 (14.86)	9 (11.54)	39 (15.92)	

Regarding the determinants of CAM usage (Table 2), the results showed high odds ratios for age, employment status, education level, and duration since diagnosis (3-10 years versus 10-20 years). However, a statistically significant result was shown in only age and employment status (student versus employee).

**Table 2:** Independent predictors of complementary and alternative medicine use in individuals with diabetes LCL: lower confidence limit; UCL: upper confidence limit

Variable	Odds ratio	LCL	UCL	P-value
Age: 18 to <65 versus ≥65 years	2.821	1.033	7.701	0.0430
Sex	0.961	0.476	1.940	0.9125
Education level: high school versus university education	0.955	0.462	1.972	0.9006
Education level: illiterate versus university education	0.682	0.215	2.163	0.5158
Education level: postgraduate versus university education	0.739	0.203	2.691	0.6467
Education level: primary/can read and write versus university education	1.348	0.513	3.543	0.5442
Employment status: retired versus employee	1.404	0.540	3.648	0.4863
Employment status: student versus employee	4.643	1.334	16.162	0.0159
Employment status: unemployed versus employee	2.370	0.897	6.264	0.0817
Marital status: divorced versus married	1.005	0.243	4.146	0.9949
Marital status: single versus married	0.417	0.132	1.320	0.1370
Marital status: widow versus married	1.261	0.430	3.696	0.6730
Diabetes type	0.669	0.250	1.794	0.4245
Duration since diagnosis: 1-3 versus 10-20 years	0.959	0.307	2.989	0.9418
Duration since diagnosis: 3-10 versus 10-20 years	1.028	0.528	1.999	0.9361
Duration since diagnosis: >20 versus 10-20 years	0.695	0.325	1.488	0.3490
Oral or injectable	0.965	0.367	2.538	0.9422
Insulin injections	0.742	0.309	1.777	0.5025

As shown in Table 3, approximately 25% of CAM users used daily treatment. Family and friends were the main sources of information for 60% of CAM users. Blood sugar control did not improve in 46% of CAM users, while 51% affirmed their blood sugar readings to be better with CAM usage. However, 83% of these patients did not change their treatment plans during CAM usage, and 62.5% did not inform their physicians about CAM use.

**Table 3:** Complementary and alternative medicine user attributes (n=89) CAM: complementary and alternative medicine

Attribute		n (%)
Frequency of CAM use	2-4 times/week	15 (18.99%)
	One month at the beginning of diagnosis	1 (1.27%)
	Daily	20 (25.32%)
	Once per week	23 (29.11%)
	Monthly	20 (25.32%)
Source of CAM information	Media and social media	29 (32.58%)
	CAM practitioner	21 (23.6%)
	Family and friends	54 (60.67%)
	Published medical research	2 (2.25%)
	Physicians	5 (5.62%)
	Religious sources	1 (1.12%)
	Commercial advertisements	4 (4.49%)
Did CAM improve diabetes control?	I have used it for a short time	1 (1.25%)
	No	37 (46.25%)
	Somewhat	1 (1.25%)
	Yes	41 (51.25%)
Did the CAM user inform his physician about using CAM?	No	50 (62.5%)
	Yes	30 (37.5%)
Did the CAM user or his physician change his prescription?	Changing the treatment plan while using CAM	1 (1.25%)
	Holding all treatments	1 (1.25%)
	No changes	67 (83.75%)
	Reducing oral agent doses	1 (1.25%)
	Reducing oral and injectable agent doses	10 (12.5%)

Most of the patients (53.93%) had used mind-body therapy such as meditation, Ruqyah (a Quran-based healing method), or relaxation techniques. Approximately 37% of CAM users used more than one type of CAM. The most commonly used combination was mind-body therapy and biologically based CAM. Black cumin was the most used herb, followed by fenugreek, myrrh, cinnamon, garlic, and onion. Approximately 5% of CAM users consumed dates to treat diabetes. Table 4 and Table 5 provide more details about the types of CAM used.

**Table 4:** Type of CAM used among CAM users (n=89) ^a^Walking barefoot on mixed soil, bee stings, drinking hot water after waking up from sleep, etc. CAM: complementary and alternative medicine

Which CAM therapies have you tried for diabetes?	Number (%)
1. Mind-body therapy (meditation, Ruqyah, relaxation)	48 (53.93%)
2. Biologically based CAM (vitamins, mineral nutritional supplements, probiotics, honey products, diet)	41 (46.07%)
3. Manipulative and body-based CAM (chiropractic, massage, reflexology, acupuncture, cupping)	12 (13.48%)
4. Others^a^	8 (8.99%)

**Table 5:** Type of herb used by complementary and alternative medicine users (n=89) Note: Some of the patients used more than one type. CAM: complementary and alternative medicine

Herb name		Local name	Number (%)
Black cumin/black seeds	Habat souda/albarakah		37 (41.57)
Fenugreek	Helbah		25 (28.09)
Myrrh	Murrah		21 (23.6)
Frankincense	Leban		20 (22.47)
Olive leave	Awraq alzaiton		18 (20.22)
Cinnamon	Gerfah		14 (15.7)
Garlic	Thom		14 (15.7)
Onion	Basal		14 (15.7)
Lupinus albus	Altarmis		9 (10.11)
Asafetida	Alhalatayt		9 (10.11)
Pomegranate	Rumaan		8 (8.99)
Barley	Shaeer		6 (6.7)
Wormwood	Sheeh		5 (5.62)
Dates	Tamer		5 (5.62)
Olive oil	Zait alzaiton		4 (4.49)
Green gourd juice	Easir alqare al’akhdar		4 (4.49)
Aloe vera	Alsabaar		3 (3.37)
Ginger	Zanjabeel		2 (2.25)
Rosemary	Ekleel aljabal		1 (1.12)
Moringa	Almurinja		1 (1.12)
Green tea	Shay ‘akhdar		1 (1.12)
Cod liver oil	Zayt kabid samak		1 (1.12)
Juniperus	Alearear		1 (1.12)
Safflower	Alqurtam		1 (1.12)
Sage	Almaryamia		1 (1.12)

## Discussion

This study showed that 26% of the patients with diabetes who attended the endocrine and diabetes clinic in King Abdulaziz Medical City used CAM; 67.5% had type 2 diabetes, and the remainder had type 1. Patients with type 2 diabetes represented 67.5% of CAM users, while patients with type 1 diabetes represented 32.5% of CAM users. The prevalence findings in this study are similar to those of two previous Saudi studies, which found that 30.5% and 33.7% of patients with diabetes used CAM [13,14]. In one study, 38.5% of patients with type 1 diabetes used herbs [15]. One literature review about CAM found that 32.2% of Saudi patients with diabetes used CAM [16]. Among Eastern Mediterranean countries, the prevalence of CAM use is between 9% and 88% [17]. One study in Pakistan found that 57.8% of patients with type 2 diabetes had used CAM therapy [18]. In one meta-analysis, the prevalence of CAM used by patients with type 2 diabetes varied from 16.6% in Jordan to 76% in Sri Lanka [19]. Our study included patients with type 2 diabetes and a good percentage of patients with type 1 diabetes (43%) and assessed them exclusively, while previous studies either mentioned diabetics in general or type 2 diabetes patients alone. The inclusion of patients with type 1 diabetes might attenuate the prevalence of CAM use since the main treatment for this type of diabetes is insulin.

The prevalence of females who used CAMs was higher than that of males, but this difference was not statistically significant. However, this discrepancy between males and females has been observed in many national and international studies [12,13,15,17,20-22]. A comparison of the utilization of other healthcare services between Saudi men and women is needed.

An age of ≥65 years and being employed were identified as the strongest predictors of CAM use in this study. These findings are supported by those of previous studies [17,22]. Al-Eidi et al. found that patients aged 51 years and older and those who are currently unemployed were most likely to use CAM [14]. Another Saudi study did not find any association between demographic data and CAM use [15]. This present study also found that the number of patients using CAM increased with diabetes duration. This finding was supported by local and regional studies [13,14,17]. Our study also found that patients with diabetic foot pathology were most likely to use CAM. However, another study found that most patients with diabetes who experienced retinopathy or other complications used CAM to mitigate the symptoms and avoid side effects [17]. The difference in predictors between studies might be related to the differences in the sample size, study method, inclusion criteria, data collection method, and different cultures.

Of our patients who used CAM, 54% underwent a form of mind-body therapy, 46% used biologically based CAM, and 13.5% used manipulative and body-based CAM. Other studies have shown that most patients used prayers, followed by herbal medicine, wet cupping, and honeybee products [7,23]. Three Saudi studies were conducted to estimate the use of herbs and traditional remedies in patients with diabetes and found that 17.4%, 30%, and 64% of them used herbs as treatment [15,24,25]. Alsanad et al. found that the most commonly used CAM was honey, followed by wet cupping and vitamins, while spiritual healing (Ruqyah) was used less frequently [16]. The difference between this study and previous studies could be explained by different methodologies and our inclusion of patients with type 1 diabetes, which has not been clearly presented in previous studies.

The most commonly used herbal products in this study were black cumin (41.6%), followed by fenugreek (28%), myrrh (23.6%), frankincense (22.4%), cinnamon (15%), garlic (15%), and onion (15%), which are similar to that reported in many studies in Saudi Arabia [15]. Of the patients in this study, 5% used dates as treatment; this was also noticed in a study by Al-Saeedi et al., where 17% believed in the medicinal properties of dates and 8.7% used dates for diabetes [25]. Dates are a part of the Saudi traditional diet. As known, medical nutrition therapy is an important part of diabetes management. Therefore, every patient should be assessed based on his/her diet and food intake and referred to a medical nutrition therapist as part of the treatment.

More than 60% of the patients in this study did not inform their physicians about their CAM use. In addition, most CAM users in this study (52.6%) had an HbA1c ≥ 9%. However, most of the non-CAM users (61.6%) had HbA1c levels <9%. In another study, more than 50% of the patients did not inform their physicians that they were using CAM [15,22]. Across the Eastern Mediterranean region, approximately 40% of CAM users did not inform their physicians. Asking patients about CAM use during every clinical encounter is important, especially if there is a change in blood sugar control or a decrease in medication dispensing. More than 80% of the patients in this study did not change their treatment plan while using CAM. However, non-compliance with diabetic medication among CAM users was noted. [18]. One study showed that 57% of CAM users were poorly adherent compared to 47% of non-CAM users, and 31% of CAM users had a history of medication discontinuation compared to 22.5% of non-CAM users [26]. One of the reasons for the non-disclosure to the physician was that the “doctor never asked” [27]. This also emphasizes the importance of good relationships between physicians and their patients, especially in patients with type 1 diabetes.

Family and friends were the main sources of CAM knowledge, as shown in many previous studies [12,14]. This is expected in highly social and connected societies, such as Saudi Arabia. In addition, there could be other reasons such as limited education in subjects such as CAMs in schools and few public health campaigns. It is important to find an easy, accessible, and updated resource about CAM to educate patients and their families and minimize misinformation.

This study has some limitations. First, it is cross-sectional and was conducted at one center, which might have affected the generalizability of the results. Data were collected using an interview, and this might have prevented some patients from disclosing information and increased recall bias, especially regarding their HbA1c level. In addition, this study did not specifically ask about the type of CAM used in each category, except in the case of biologically based CAM.

This study suggests that more studies including patients with type 1 diabetes are required. More studies are needed to understand the extent and type of CAM use and determine if this has an effect on HbA1c values. Since emerging types of CAM exist, investigating each type of CAM independent of other types is warranted to provide more accurate results regarding the prevalence and effect of each CAM type on diabetes.

## Conclusions

The use of CAM is common in our society and can affect some patients’ adherence to medications. Good communication with diabetic patients and assessing CAM use are essential. In this study, females used CAM more frequently than males. However, older age and employment status were the most likely predictors of CAM use. The main source of information for CAM use is from family and friends. Therefore, it would be helpful to have additional validated information and studies readily accessible to patients concerning the use of CAM as an additional tool to control diabetes. Further studies on each type of CAM are advised, and directing patients and the community to a valid source of CAM is essential.
